# Active Fraction Combination from Liuwei Dihuang Decoction (LW-AFC) Alleviated the LPS-Induced Long-Term Potentiation Impairment and Glial Cells Activation in Hippocampus of Mice by Modulating Immune Responses

**DOI:** 10.1155/2019/3040972

**Published:** 2019-09-16

**Authors:** Ju Zeng, Bin Cheng, Yan Huang, Xiaorui Zhang, Chen Wang, Na Sun, Gang Liu, Xiaorui Cheng, Yongxiang Zhang, Wenxia Zhou

**Affiliations:** ^1^Beijing Institute of Pharmacology and Toxicology, Beijing 100850, China; ^2^State Key Laboratory of Toxicology and Medical Countermeasures, Beijing 100850, China

## Abstract

Neuroinflammation is known as a typical feature associated with many neurodegenerative diseases including Alzheimer's disease (AD) and impairs the synaptic plasticity of the hippocampus. LW-AFC is an active fraction combination being extracted from Liuwei Dihuang decoction, a classic traditional Chinese medicine prescription. This study aimed to investigate the effects of LW-AFC on synaptic plasticity in mice with lipopolysaccharide (LPS) treatment. The results showed that the administration of LPS caused fever and long-term potentiation (LTP) impairment in mice. The pretreatment with LW-AFC had an antipyretic effect on fever and improved the impaired LTP induced by LPS, alleviated the microglia and astrocytes activation in the hippocampus, regulated the abnormal T-lymphocyte subpopulation in the spleen and blood caused by LPS, and reduced the aberrant secretion of cytokines in the brain and plasma. The compounds paeoniflorin, morroniside, and loganic acid in LW-AFC regulated the TNF-*α* secretion in non-LPS- and LPS-stimulated BV-2 cells. These data suggest that LW-AFC improves the LPS-induced impairment of LTP and alleviates the activation of glial cells in the hippocampus, which might be associated with modulating immune responses.

## 1. Introduction

Neuroinflammation is known as a typical feature associated with many neurodegenerative diseases, including Alzheimer's disease (AD) [1–7]. Neuroinflammation is noteworthy because increased inflammation is harmful to the local brain environment and produces synaptoxic and neurotoxic effects [8, 9] associated with cognitive impairment [10, 11]. Growing evidence indicates that systemic administration of lipopolysaccharides (LPSs) stimulates the inflammatory response in the brain [12–14]. This is a commonly used model of neuroinflammation, which leads to an increased secretion of proinflammatory cytokines like tumor necrosis factor alpha (TNF-*α*), interleukin-1 (IL-1), and interleukin-6 (IL-6) in the brain immune system [15–17]. The elevated cytokine levels mediate sickness behavior and alter cognitive processes accompanied by the occurrence of neuroinflammation [14, 18]. The inflammatory response impairs the synaptic plasticity of the hippocampus [13, 19].

Reducing inflammation is one of the most effective treatment strategies to improve cognitive impairment. Selective cyclooxygenase-2 (COX-2) inhibitors, like celecoxib, ibuprofen, and indomethacin, whose anti-inflammatory and analgesic effects partly result from the inhibition of prostaglandin synthesis, are clinically used to treat pain and arthritis. In subsequent years, a host of experimental studies have reported the A*β*-lowering effects and cognitive improvement of these drugs in cell-based and animal models of Alzheimer's disease or clinical trials [20, 21]. Proinflammatory cytokine tumor necrosis factor alpha (TNF-*α*) inhibitor etanercept has been used in clinical research and is expected to inhibit the activation of microglia by its anti-inflammatory action to improve the cognitive effect of AD patients [22, 23].

LW-AFC is a new formula derived from the Liuwei Dihuang decoction, which is a classic traditional Chinese medicine prescription. LW-AFC is composed of glycosides, oligosaccharides, and polysaccharides. Our previous studies have shown that LW-AFC has good immunological activity [24] and can improve cognitive performance in sporadic and family AD model mice, such as the senescence-accelerated mouse prone 8 strain (SAMP8) and APP/PS1 transgenic mice [25, 26]. The impairments in long-term potentiation (LTP) were significantly improved by LW-AFC administration in SAMP8 mice and in the corticosterone-induced LTP inhibition mice model [27]. LW-AFC can significantly reduce A*β* plaque aggregation in the brain of APP/PS1 transgenic mice [25]. All these studies imply that LW-AFC is a promising effective drug for AD treatment; however, whether LW-AFC has any therapeutic effect on LPS-treated mice and the underlying mechanisms remain unclear, thus warranting further investigation. The present study aimed to investigate the effects of LW-AFC on the synaptic plasticity and the immunomodulatory effects of LW-AFC on the inflammatory responses in LPS-treated mice in order to elucidate whether LW-AFC could improve synaptic plasticity impairment by regulating immune dysfunction.

## 2. Materials and Methods

### 2.1. Animals

A total of 70 male 2–4 months BALB/c mice were purchased from Weitong Lihua Experimental Technology Co. Ltd. (Beijing, China). Mice weighing 19–21 g were housed in cages maintained at 23 ± 1°C with food and water and a 12 : 12 h dark/light cycle. Mice were acclimatized to the laboratory environment for at least one week prior to the experiment. The animal received human care according to the National Institutes of Health (USA) guidelines, approved by the Institute of Animal Care and Use Committee (IACUC) of the National Beijing Center for Drug Safety Evaluation and Research (NBCDSER) (No. 2018-030).

### 2.2. Drug and Reagents

The origin herbal drug of LW was purchased from Beijing Tongrentang Co., Ltd. (Beijing, China). LW-AFC is comprised of glycosides, oligosaccharides, and polysaccharides extracted from LW. Details of the extraction of LW-AFC can be seen in [26]. We chose indomethacin (≥98%, Ouhe Technology Co., Ltd., Beijing, China) as a compound that has a positive effect on the inflammatory response. Lipopolysaccharides (L2880, Sigma, St. Louis, MO, USA), PEG400 (30150892, Sinopharm Group Chemical Reagent Co., Ltd. Shanghai, China), mouse cytokines/chemokines kit (9MPXMCYTO-70K-06, Merck Millipore, Boston, MA, USA), BD FACS™ Lysing Solution (349202, BD), 4% paraformaldehyde, saline, 75% alcohol, 4% EDTA-Na2, and PBS.

### 2.3. Drug Administration

LW-AFC was intragastrically administered in 0.8, 1.6, or 3.2 g/kg doses for 14 days. 2.5 mg/kg indomethacin was used as the positive drug with a single intraperitoneal (i.p.) injection on the last day. All drugs were administered in an equivalent volume of 0.1 mL/10 g body weight of the mouse. Model group and drug groups were intraperitoneally administered LPS 0.25 mg/kg 30 min after being given the drug on the 14th day.

### 2.4. Temperature Test

We employed an electronic thermometer to measure the neck temperature of the mice 6 h after the LPS i.p. injection.

### 2.5. In Vivo LTP Test

Mice were anesthetized with urethane 1.5 g/kg i.p., and then we used the stereotaxic apparatus (SR-6N, Narishige Inc., Tokyo, Japan) to fix the mouse. The mouse scalp was cut, the hippocampal PP-DG (anterior penetrating fiber-dentate granule cell layer) position was located, a hole was drilled at the localization, the recording electrode was inserted into the DG (2.0 mm after the anterior fontanel, 1.4 mm beside the midline, and 1.5 mm under the subdural layer), the stimulating electrode was inserted into the PP (3.8 mm after the anterior fontanel, 3.0 mm beside the midline, and 1.5 mm under the subdural layer), and the reference electrode was clamped on the scalp. During the experiment, the environment was kept quiet. A population spike (PS, bandwidth: 100 *μ*s, current intensity: 0.3 mA) was conducted, and the electrodes were modulated to achieve the best PS wave shape. After 30∼60 min, the stimulating intensity was decreased to half the PS wave and then was kept stable for 30 min and recorded as the baseline. Then, tetanic stimulation (TS) was given to induce long-term potentiation (LTP) and recode the PS wave for 60 min.

### 2.6. Immunofluorescence Staining

Animals were sacrificed after the LTP test. Brains were subsequently removed from the skull after decapitation and perfused with PBS. Each brain was cut into two hemispheres. Tissues of the hippocampus and cortex in one hemisphere were separated and then frozen in liquid nitrogen for detecting the concentration of cytokines. The other hemisphere was immersion fixated in 4% paraformaldehyde, embedded in paraffin, and then cut into 5 *μ*m thick sections for immunofluorescence staining. The sections were incubated in 2% fetal bovine serum for 1 h and then were incubated with the primary antibodies of rabbit anti-Iba1 (1 : 500; Abcam, Cambridge, UK) and rabbit anti-GFAP (1 : 1000; Abcam, Cambridge, UK) to label the microglia and astrocytes at 4°C overnight. Then, they were incubated with the appropriate secondary antibody (goat anti-rabbit, 1 : 1000; Zhongshan Jinqiao, Beijing, China) for 2 h at room temperature. Images were captured using a Pannoramic 250/MIDI camera (3D HISTECH, Budapest, Hungary) and analyzed using Image-pro plus 6.0 software.

### 2.7. Flow Cytometry Analysis

The spleen single-cell suspension was prepared by 40 *μ*M nylon cell strainer, and the red blood cells were depleted in a Tris-NH_4_Cl lysis buffer (0.017 M Tris-HCl, 0.144 M NH_4_Cl). The cells were incubated with FITC anti-mouse CD3, Percp anti-mouse CD4, APC anti-mouse CD25, APC anti-mouse CD8 (BioLegend, San Diego, CA, USA) at room temperature for 30 min in the dark and then washed and resuspended in 0.5 mL of PBS containing 0.5% FBS and then tested by cytometry (BD Calibur™, company, Lake Franklin, NJ, USA). The percentages of CD3^+^, CD3^+^CD4^+^, CD3^+^CD25^+^, and CD3^+^CD8^+^ expression was analyzed by the BD FACS Calibur™ CellQuest software.

### 2.8. Measurement of Cytokine Concentration

The blood was dropped into a tube containing 4% EDTA-Na_2_ (1 : 9), placed at 4°C for 2 h, centrifugated at 4°C, 3000 ×*g* for 15 min, and then the plasma was collected into clean tubes and stored at −70°C for the cytokine test. The concentrations of TNF-*α*, IFN*γ*, IL-1*β*, IL-6, IL-10, IL-12, MCP-1, and G-CSF were detected by a Multiplex Map Kit (MCYTOMAG-70K, Merck Millipore, Boston, MA, USA) according to the manufacturer's instructions and analyzed with Millipore software (Merck Millipore Corp., Boston, MA, USA).

### 2.9. Cell Culture and Stimulation

Mouse microglia BV-2 cells were cultured in high-glucose Dulbecco's Modified Eagle's Medium (DMEM) supplemented with 10% fetal bovine serum (FBS, Gibco, Grand Island, NY, USA), 1% penicillin, and streptomycin at 37°C in a humidified incubator with 95% air and 5% CO_2_. Cells were seeded at 1 × 10^4^/well in a 96-well plate. When cells reached approximately 70% confluence, we removed the culture medium and pretreated them with 1, 10, and 100 *μ*M paeoniflorin, morroniside, loganic acid, stachyose, and mannotriose (J&K Chemical, Shanghai, China) and 0.1, 1, and 10 *μ*M CA4-3 [12] for 30 min before adding 1 *μ*g/mL LPS stimulate (L2630, Sigma, St. Louis, MO, USA) and then incubated for 24 h. We used 25 *μ*M indomethacin and 10 *μ*M dexamethasone as a positive control.

### 2.10. Cell Viability Assay

After the cells were cultured for 24 h, the cell viability was examined by a Cell Counting Kit 8 (Dojindo Laboratories, Kumamoto, Japan), according to the manufacturer's instructions. We added 10 *μ*L of CCK-8 to each well and then incubated them at 37°C for 2 h. The optical density (OD) was measured using an EnSpire™ 2300 instrument (PerkinElmer, Waltham, MA, USA) at 450 nm.

### 2.11. Measurement of TNF-*α* Secretion

The secretion of TNF-*α* in the cell supernatant was determined using an AlphaLISA mouse TNF-*α* Kit (PerkinElmer, Waltham, MA, USA) according to the manufacturer's protocols using an EnVision™ 2104 instrument (PerkinElmer, Waltham, MA, USA).

### 2.12. Statistical Analysis

All data were analyzed using GraphPad Prism 6.0 and are expressed as mean ± SD. Statistical significance was determined by unpaired Student's *t*-test and one-way ANOVA and Dunnett's test. *p* values <0.05 were considered significant.

## 3. Results

### 3.1. Antipyretic Effect of Pretreatment of LW-AFC on Fever Caused by Intraperitoneal Injection of LPS

**Figure 1:**
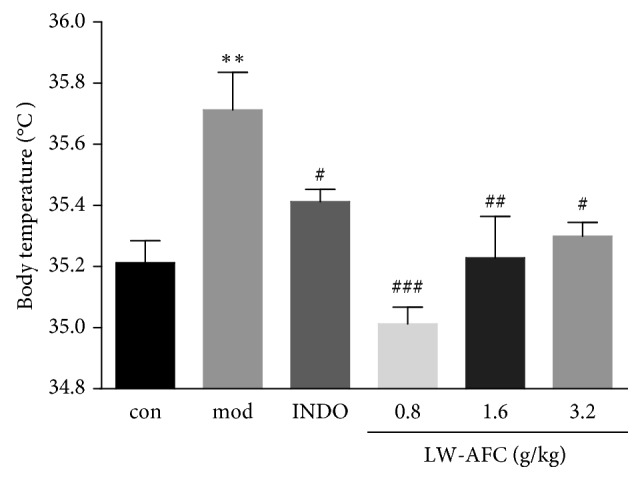
Effect of LW-AFC on body temperature in LPS-treated mice. ^*∗∗*^*p* < 0.01*vs*. con and Student's *t*-test; ^###^*p* < 0.001, ^##^*p* < 0.01, and ^#^*p* < 0.05*vs*. mod, one-way ANOVA, and Dunnett's test, mean ± SD, *n* = 8. con, control; mod, model; INDO, indomethacin.

Fever is a feature of inflammation-induced symptoms. The intraperitoneal injection of LPS can trigger systemic inflammation that results in fever. In this study, to confirm that LPS induced a sickness response and investigate the effect of LW-AFC on this sickness, we measured the change in body temperature. The results showed that the body temperature in mice rose significantly after 6 h of a single LPS injection (Figure 1). Pretreatment with 30 min of indomethacin and two weeks of LW-AFC prevented the increase in temperature caused by the intraperitoneal (i.p.) injection of LPS. LW-AFC had a significant dose-dependent effect on reducing fever (Figure 1).

### 3.2. Pretreatment of LW-AFC Improved Long-Term Potentiation Impairment Induced by Intraperitoneal Injection of LPS

**Figure 2:**
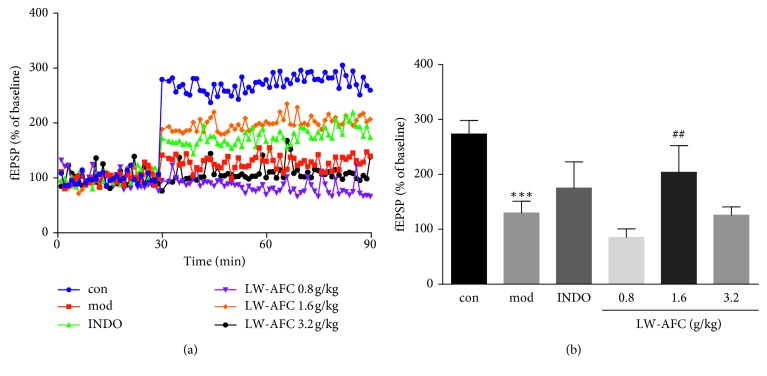
Effect of LW-AFC on LTP impairment in the hippocampus of LPS-treated mice. TS was given after 30 min baseline recording. (a) The time course of the indicated dose of LW-AFC on the population spike. (b) The average population spike in 60 min of each group. ^*∗∗∗*^*p* < 0.001*vs*. con and Student's *t*-test; ^##^*p* < 0.01*vs*. mod, one-way ANOVA, and Dunnett's test, mean ± SD, *n* = 3–5. con, control; mod, model; INDO, indomethacin.

In order to understand the effect of LW-AFC on the cognitive change of LPS-induced mice, we firstly conducted the object recognition behavior test to investigate the temporal order memory in LPS-injected mice, but there were no significant differences in LW-AFC treatment (Supplementary [Supplementary-material supplementary-material-1]). LTP is associated with synaptic plasticity and is known as the physiological mechanism of learning and memory in the CNS [28, 29]. In this study, we found that the i.p. injection of LPS in mice (mod) caused a significant decrease in the EPSP wave when compared to the control group (con) (Figure 2, [Supplementary-material supplementary-material-1]). This suggests that system injection of LPS could induce neuroinflammation, which can impair learning and memory. Compared with the model group (mod), pretreatment with LW-AFC had a significant increasing effect on LTP at a dose of 1.6 g/kg. The data suggest that pretreatment with LW-AFC might improve the ability of learning and memory in mice treated with an intraperitoneal injection of LPS.

### 3.3. Pretreatment of LW-AFC Alleviated Activation of Glial Cells in the Brain of Mice with Intraperitoneal Injection of LPS

**Figure 3:**
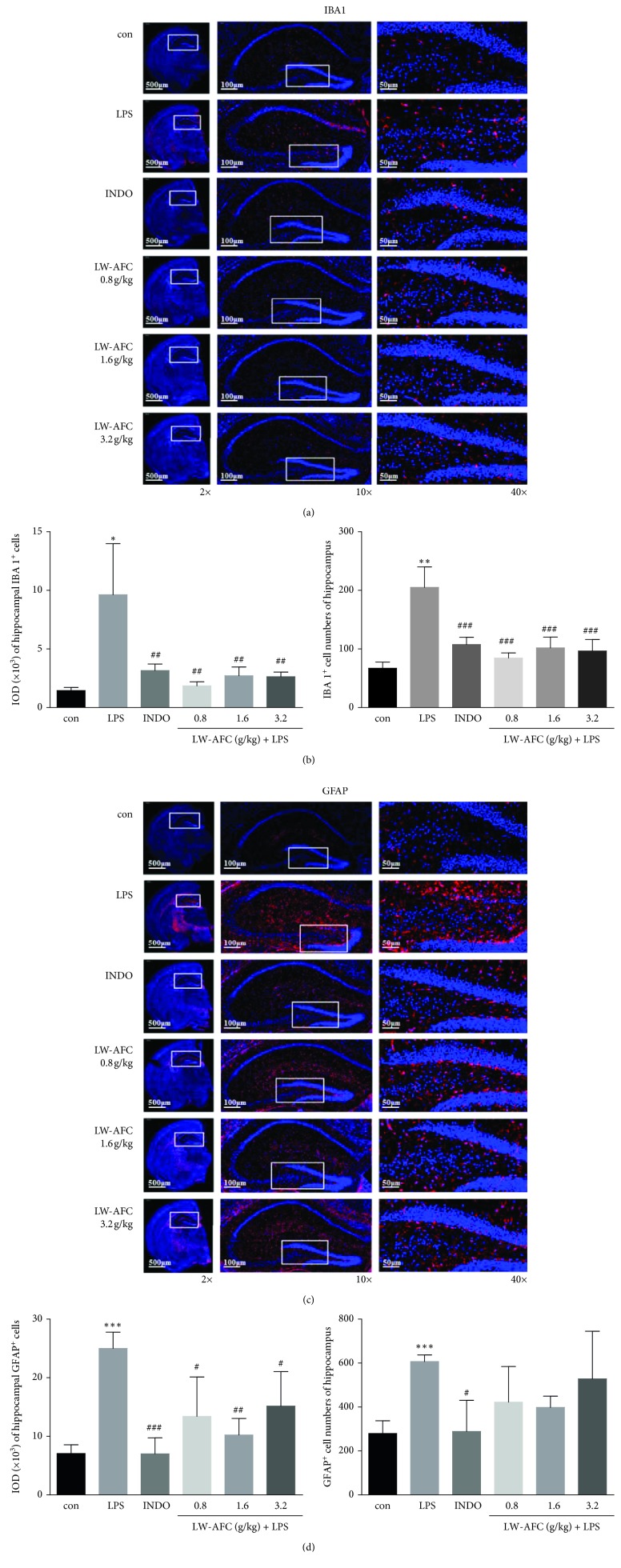
Effect of LW-AFC on microglia and astrocytes activation in the brain of LPS-induced mice. (a, c) Representative images of IBA1-labeled microglia and GFAP-labeled astrocytes in the brain and hippocampus, and activated glia is shown in red. 2x, scale bars = 500 *μ*m; 10x, scale bars = 100 *μ*m; 40x, scale bars = 50 *μ*m. (b, d) Quantification of the IOD (integrated optical density) value and the IBA1+ and GFAP + cells numbers. ^*∗∗*^*p* < 0.01 and ^*∗*^*p* < 0.05*vs*. con and Student's *t*-test; ^###^*p* < 0.001 and ^##^*p* < 0.01*vs*. mod, one-way ANOVA, and Dunnett's test. Mean ± SD, *n* = 3. con, control; mod, model; INDO, indomethacin.

Activation of the microglia and astrocytes in the brain is a typical feature of inflammation in the CNS and one of the causes of impairment of learning and memory. We found that the administration of a single LPS intraperitoneal injection significantly induced the activation of microglia (Figure 3(a)) and astrocytes (Figure 3(c)) in the brains of mice. Pretreatment with indomethacin or LW-AFC attenuated the activation of LPS-induced microglial cells (Figure 3(b)) and astrocytes (Figure 3(d)) in the hippocampus of mice.

### 3.4. Pretreatment of LW-AFC Modulated the Abnormality of Lymphocyte Subpopulations and Cytokine Secretion Caused by Intraperitoneal Injection of LPS

**Figure 4:**
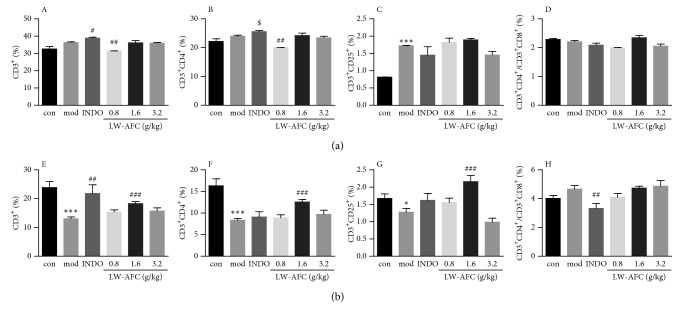
Effect of LW-AFC on lymphocyte subpopulations in the (a) spleen and (b) blood of LPS-treated mice. ^*∗∗∗*^*p* < 0.001, ^*∗∗*^*p* < 0.01, and ^*∗*^*p* < 0.05*vs*. con and student's *t*-test; ^###^*p* < 0.001, ^##^*p* < 0.01, and ^#^*p* < 0.05*vs*. mod, one-way ANOVA, and Dunnett's test. (A–D) Percentage of lymphocyte subpopulations in the spleen, mean ± SD, *n* = 3. (E–H) Percentage of lymphocyte subpopulations in the blood, mean ± SD, *n* = 8. con, control; mod, model; INDO, indomethacin.

To figure out the immunomodulatory effects of LW-AFC on LPS-induced inflammation, we detected the contents of lymphocyte subpopulations in the spleen and peripheral blood and the concentration of cytokines in the hippocampus and plasma. The results showed that intraperitoneal injection of LPS caused an increase in the activated T-cell CD3^+^CD25^+^ (Figure 4(c)) in the spleen and T-cell CD3^+^ ((Figure 4(a)), T-helper cell CD3^+^CD4^+^ (Figure 4(b)), and CD3^+^CD4^+^/CD3^+^CD8^+^ (Figure 4(d)) did not change. In blood, it reduced the expression of T-cell CD3^+^ (Figure 4(e)), CD3^+^CD4^+^ (Figure 4(f)), and CD3^+^CD25^+^ (Figure 4(g)) and a little increase of the CD3^+^CD4^+^/CD3^+^CD8^+^ (Figure 4(h)). Pretreatment with LW-AFC reversed the abnormality of some lymphocyte subpopulations caused by the intraperitoneal injection of LPS: CD3^+^CD4^+^ (Figure 4(b)) in the spleen and CD3^+^ (Figure 4(e)), CD3^+^CD4^+^ (Figure 4(f)), and CD3^+^CD25^+^ (Figure 4(g)) in the blood.

**Figure 5:**
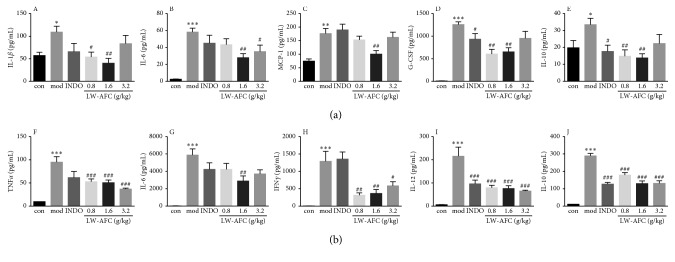
Effect of LW-AFC on cytokine secretion in the (a) hippocampus and (b) plasma of LPS-treated mice. ^*∗∗∗*^*p* < 0.001, ^*∗∗*^*p* < 0.01, and ^*∗*^*p* < 0.05*vs.* con and Student's *t*-test; ^###^*p* < 0.001, ^##^*p* < 0.01, and ^#^*p* < 0.05*vs*. mod, one-way ANOVA, and Dunnett's test, mean ± SD, *n* = 5–8. A, IL-1*β*; B, IL-6; C, MCP-1; D, G-CSF; E, IL-10; F, TNF-*α*; G, IL-6; H, IFN*γ*; I, IL-12; J, IL-10. con, control; mod, model; INDO, indomethacin.

The administration of an intraperitoneal injection of LPS increased the secretion of proinflammatory factor IL-6 in the hippocampus (Figure 5(b)) and plasma (Figure 5(g)) and also induced the increase in proinflammatory factor IL-1*β* (Figure 5(a)) and MCP-1 (Figure 5(c)) in the hippocampus; anti-inflammatory cytokine G-CSF (Figure 5(d)) and IL-10 (Figure 5(e)) in the hippocampus; proinflammatory factor TNF-*α* (Figure 5(f)), IFN-*γ* (Figure 5(h)), and IL-12 (Figure 5(i)) in plasma; and anti-inflammatory cytokine IL-10 (Figure 5(j)) in plasma. The data indicate that pretreatment with LW-AFC reverses the aberrant secretion of cytokine including proinflammatory factor IL-1*β* (Figure 5(a)), IL-6 (Figure 5(b)), and MCP-1 (Figure 5(c)) in the hippocampus; anti-inflammatory cytokine IL-10 (Figure 5(e)) and G-CSF (Figure 5(d)) in the hippocampus; proinflammatory factor TNF-*α* (Figure 5(f)), IL-6 (Figure 5(g)), IFN-*γ* (Figure 5(h)), and IL-12 (Figure 5(i)) in plasma; and anti-inflammatory cytokine IL-10 (Figure 5(j)) in plasma. We also detected the cytokines in the cortex; LW-AFC only decreased the level of MCP-1 in the cortex ([Supplementary-material supplementary-material-1]) and had no significant effect on other cytokines.

### 3.5. Effect of LW-AFC Compounds on Cell Viability and Secretion of TNF-*α* in LPS-Stimulated BV-2 Cells

**Figure 6:**
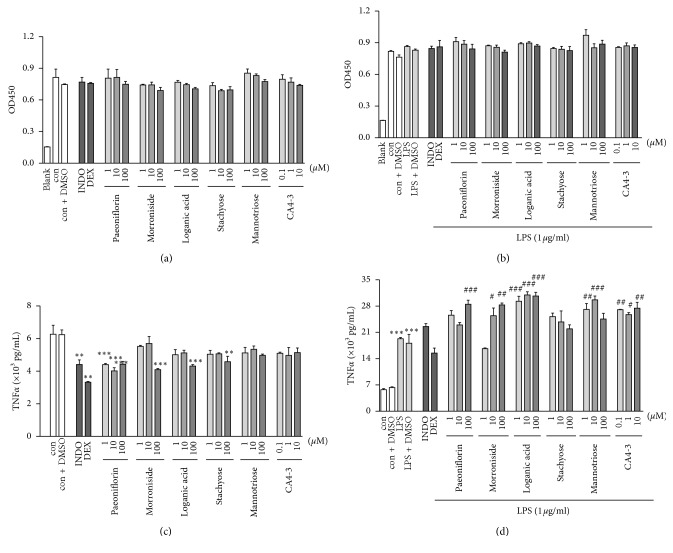
Effect of compounds from LW-AFC on cell viability and secretion of TNF-*α* in LPS-stimulated and non-LPS-stimulated BV-2 cells: (a) the cell viability of non-LPS-stimulated BV-2 cells; (b) the cell viability of LPS-stimulated BV-2 cells; (c) secretion of TNF-*α* in non-LPS-stimulated BV-2 cells; (d) secretion of TNF-*α* in LPS-stimulated BV-2 cells. ^*∗∗∗*^*p* < 0.001 and ^*∗∗*^*p* < 0.01*vs*. con and Student's *t*-test; ^###^*p* < 0.001, ^##^*p* < 0.01, and ^#^*p* < 0.05, *vs*. mod, one-way ANOVA, and Dunnett's test. Mean ± SD *n* = 3. con, control; mod, model; INDO, indomethacin; DEX, dexamethasone.

Microglia are important immune cells in the CNS. TNF-*α* is the earliest and most important inflammatory mediator in the process of inflammation, not only promoting the occurrence of inflammation but also a key physiological regulator of hippocampal synaptic function [30]. Therefore, to further study the immunomodulatory effect of LW-AFC, we evaluated the effects of representative compounds from glycosides, oligosaccharides, and polysaccharides—the three active fractions of LW-AFC on BV-2 cells. The data show that paeoniflorin, morroniside, and loganic acid from the glycosides; stachyose and mannotriose from the oligosaccharides; and CA4-3 from the polysaccharides did not result in any cytotoxic effects on non-LPS-stimulated (Figure 6(a)) and LPS-stimulated BV-2 cells (Figure 6(b)). Pretreatment with paeoniflorin at dosage of 1 *μ*M, 10 *μ*M, and 100 *μ*M, morroniside, loganic acid, and stachyose, respectively, at a dosage of 100 *μ*M significantly reduced the TNF-*α* secretion in non-LPS-stimulated BV-2 cells (Figure 6(c)). At dosage of 0.1 *μ*M, only the pretreatment with CA4-3 increased TNF-*α* secretion of BV-2 cells stimulated by LPS. At dosage of 1 *μ*M, loganic acid, mannotriose, and CA4-3, respectively, increased TNF-*α* secretion. At dosage of 10 *μ*M, morroniside, loganic acid, mannotriose, and CA4-3 had the effect of increasing TNF-*α* secretion. At dosage of 100 *μ*M, paeoniflorin, morroniside, and loganic acid increased of TNF-*α* secretion of BV-2 cells stimulated by LPS (Figure 6(d)).

## 4. Discussion

The results suggest that LW-AFC improves the LPS-induced inhibition of LTP in the hippocampus, reduces the activation of glial cells, and regulates the abnormality of cytokines and lymphocyte subtypes, which have good anti-neuroinflammatory activation. Under normal physiological conditions, the immune response is beneficial for regulating the remodeling of neural circuits, promoting memory consolidation, hippocampal LTP, and neurogenesis, which are mediated by complex interactions among the brain cells with microglia and astrocytes, peripheral immune cells, in particular T cells and macrophages, and neurons [31, 32]. Proinflammatory cytokines disrupt the delicate balance needed for the neurophysiological actions of immune processes and produce direct detrimental effects on memory and synaptic plasticity [33]. LTP is one of the most widely used models for studying synaptic plasticity, and it is a key cellular process in learning and memory. Both central and peripheral administration of LPS can lead to cognitive impairment [34, 35] and synaptic plasticity inhibition [36–38]. In this study, the dosage of LPS was used by referencing the literature [34], and the results showed that LTP was significantly inhibited after the i.p. injection of LPS, in line with the results reported in previous studies [37, 39]. Pretreatment with LW-AFC could improve impaired LTP induced by LPS. Evidence has shown that acute neuroinflammation impairs context discrimination memory via the disruption of the pattern separation process at the neural circuit activity level in the CA3 and CA1 of LPS-treated rats, showing strong retrieval deficits of context discrimination at the behavioral level [14, 40]. In our experiment, we performed whether the temporal order memory was impaired in LPS-injected mice. Evidence has shown that temporal order memory tested in a spatial navigation task may provide a selective behavioral marker of Alzheimer's disease [41]. Our results showed that the LW-AFC pretreatment only has tendency to improve the short-term memory impairment in our behavioral test, and this might be partially due to the duration and dosage of drug administration. However, LTP is more sensitive than the behavioral test, LW-AFC could significantly improve the LPS-induced impairment of LTP at the dosage of 1.6 g/kg LW-AFC administration, and it suggests that short-term administration of LW-AFC can significantly improve the inhibition of LTP, but it may take long duration for LW-AFC to work at the behavioral level. Besides, excessive inflammatory responses in the cortex also could lead to behavioral impacts [42], and our results showed that the effect of LW-AFC on the secretion of inflammatory factors in the hippocampus was better than that in the cortex, which might be another reason why LW-AFC did not work significantly in this performance. Fever is one of the most common symptoms accompanying inflammation, so we examined changes in body temperature in mice after acute LPS injection. The results indicated that LW-AFC had a good antipyretic effect on fever caused by inflammation; however, the effect of LW-AFC on fever at 0.8 g/kg was better than 1.6 and 3.2 g/kg and has a better effect at 1.6 g/kg on LTP, and the reason might be LW-AFC at the different dosages has different effects. The pharmacodynamics of some traditional Chinese medicines do not follow the concentration-dependent curve, like sometimes the pharmacological effect decreases with the dose increasing to a certain extent. Fever and LTP are two different events; of course, fever can affect LTP and they are related but involve different mechanisms and different pathways. LW-AFC at different dosages may have different effects on different symptoms and also act in different ways. In our study, 1.6 g/kg LW-AFC has a better effect on LTP, and our previous studies showed that LW-AFC improved cognitive performance in sporadic and family AD model mice at this dose [25, 26] and also LW-AFC at this dosage significantly ameliorated the corticosterone-induced LTP inhibition mice model and the LTP impairment in SAMP8 mice, respectively [27], so we recommend 1.6 g/kg as the optimal dose of LW-AFC, and this dosage is equivalent to 10 g/kg Liuwei Dihuang decoction for humans in the clinical effective range.

Glial cells play important roles in various physiological and pathophysiological functions [43]. Microglia are the only nucleus phagocytes within the CNS that are important to the CNS immune response. The immune defense and maintenance of neuronal function are the two main functions within the CNS [44]. Microglia-derived molecules regulate synaptic connectivity including the regulation of synaptogenesis, synaptic pruning, axon outgrowth, synapse maturation, basal synaptic transmission, and functional synapse plasticity [45–47]. Microglia activation not only induces the production of cytokines but also cross talks with astrocyte activation [48]. Reactive astrocytes are abundant in various human neurodegenerative diseases and lose most normal astrocytic functions; activated microglia induce reactive astrocytes by secreting cytokines [48]. Previous studies demonstrated that systemic LPS injection can activate microglia and consequently induce proinflammatory cytokine secretion within six hours via the NFκB pathway in the mouse hippocampus [49–51]. Our results are in line with previous reports, where the inflammatory factors IL-1*β*, IL-6, and MCP-1 were significantly elevated after LPS treatment, and such changes were accompanied by hippocampal LTP impairment or neuronal injury [52]. LW-AFC could minimize LPS-induced inflammatory mediators in the hippocampus that are secreted by activated glial cells, and this may be related to improving the inhibition of synaptic plasticity in LPS-treated mice.

Immune cells reach the CNS via blood and the meningeal lymphatic vessels, which was discovered [53]. Activated Th cells can simultaneously produce multiple cytokines, and some of the inflammatory cytokines are closely related to learning and memory, especially in the hippocampus [54, 55], where evidence has shown that cognitive functions should be impaired in the absence of T cells [56]. Many cytokines, like IL-1 and IL-6, are involved both in the genesis of pathogenic Th cells and their maintenance and act in both the peripheral immune and CNS [33, 57]. TNF acts synergistically with IFN-*γ* to promote the expression of MHC molecules on astrocytes and oligodendrocytes [58], which may increase their susceptibility to CD8^+^ T-cell-mediated cytotoxicity. Our data showed that pretreatment with LW-AFC reverses the decrease in T-helper cell CD3^+^CD4^+^ and B-cell CD19^+^ in the spleen; T-cell CD3^+^, T-helper cell CD3^+^CD4^+^, and activated T-cell CD3^+^CD25^+^ in blood caused by LPS; reduced the proinflammatory cytokine TNF-*α*, IL-6, IL-12, INF-*γ*, and the anti-inflammatory cytokine IL-10 in plasma, suggesting that LW-AFC has a good immunoregulation effect on systemic inflammatory responses.

Cytokines can modulate synaptic transmission via glia-neuron signaling [19] and are fundamental in the dynamic interaction between neurons, glia, endothelial cells, and lymphocytes. The increased level of IL-1*β* is the driving force in cognitive dysfunction pathogenesis [59, 60]. IL-1*β* is the main final effector of the inflammation-induced deficits in both LTP and memory [54, 61], and LW-AFC seems to have a strong inhibitory effect on IL-1*β* in the hippocampus. IL-12 is produced by antigen-presenting cells and B cells, which are a heterodimeric form of proinflammatory cytokines. It is related to the amyloid load in AD pathology, and inhibition of the IL-12/IL-23 pathway may attenuate pathology and improve cognitive behavior [62]. IL-10 generally acts as an anti-inflammatory factor, but IL-10 overexpression can reduce microglial phagocytosis, leading to A*β* aggregation and worsening cognitive behavior in APP mice. Blocking the IL-10 anti-inflammatory response is beneficial for rebalancing the innate immunity [63, 64]. Clinical evidence suggests that significantly increased MCP-1 levels are associated with AD pathological changes and involved in memory dysfunction [65, 66]. Pretreatment with LW-AFC reduced the aberrant secretion of proinflammatory factor MCP-1 in the cerebral cortex, IL-1*β*, IL-6, and MCP-1 in the hippocampus, as well as the anti-inflammatory cytokine IL-10 and G-CSF in the hippocampus.

Moreover, we found that LW-AFC has a good regulatory effect on neuroinflammation, and we hope to further explore the important material basis for LW-AFC. Therefore, we preliminarily evaluated the immune activity of some compounds of LW-AFC *in vitro* experiments. It is well known that the compatibility of traditional Chinese medicines can produce special effects different from a single Chinese medicine. Some traditional Chinese medicine has a bidirectional regulation effect, and the pharmacological effects are not concentration/dose-dependent. We found that the individual compounds at high dose decreased the secretion of TNF-alpha in non-LPS-stimulated BV-2 cells but increased the secretion of TNF-alpha in LPS-stimulated BV-2 cells; the results were caused partly by the compounds that have different effects at different concentrations when used alone, and they may have superposition or antagonistic effect when they interact with other compounds. The data showed that compounds have different effects on TNF-*α* secreted by non-LPS- and LPS-stimulated cells, which might be instructive for future research. Because the role of cytokines is extremely complex in the regulation of neuroimmune and neurodegenerative diseases, additional investigation is necessary to understand its cellular and molecular mechanisms, and understanding the dynamic interaction between LW-AFC and cytokines or glial cells and how they affect cognitive processes has many clinical implications.

## 5. Conclusions

Taken together, the results of the present study for the first time showed that pretreatment with LW-AFC improves the impaired LTP and alleviates the activation of microglia and astrocytes in the hippocampus of mice induced by LPS. The pretreatment of LW-AFC modulated the abnormality of lymphocyte subpopulations including T cells, T-helper cells, activated T cells, and the aberrant secretion of proinflammatory and anti-inflammatory factors caused by LPS. These findings suggest that LW-AFC improves the impaired synaptic plasticity, and activated glial cells in the hippocampus induced by LPS is possibly associated with modulating the levels of cytokines secreted in the hippocampus and blood and regulating the lymphocyte subpopulations in blood, which might be part of the mechanism of LW-AFC improves cognitive function. Therefore, LW-AFC might be a potential therapeutic agent for neuroinflammation.

## Data Availability

The data used to support the findings of this study are included within the article and the supplementary information file.

## Supplementary Materials

Supplementary Materials Figure S1: the effects of LW-AFC on the temporal order memory test in LPS-treated mice. Figure S2: the effects of LPS at different doses on long-term potentiation in mice. Figure S3: the schematic diagram of LW-AFC preparation. Figure S4: the HPLC fingerprint of LW-AFC. Figure S5: the effect of LW-AFC on cytokine secretion in the cerebral cortex of mice with intraperitoneal injection of LPS.
