# Supplementary material for: Active Fraction Combination from Liuwei Dihuang Decoction (LW-AFC) Alleviated the LPS-Induced Long-Term Potentiation Impairment and Glial Cells Activation in Hippocampus of Mice by Modulating Immune Responses
Source: Evid Based Complement Alternat Med. 2019 Sep 16;2019:3040972. doi: 10.1155/2019/3040972 (PMC6766147; doi:10.1155/2019/3040972)
Supplement: Supplementary Materials — Figure S1: the effects of LW-AFC on the temporal order memory test in LPS-treated mice. Figure S2: the effects of LPS at different doses on long-term potentiation in mice. Figure S3: the schematic diagram of LW-AFC preparation. Figure S4: the HPLC fingerprint of LW-AFC. Figure S5: the effect of LW-AFC on cytokine secretion in the cerebral cortex of mice with intraperitoneal injection of LPS. [file 3040972.f1.pdf]

Supplementary Materials

Active Fraction Combination from Liuwei Dihuang Decoction (LW-AFC), alleviated the LPS-induced Long-term potentiation impairment and glial cells activation in hippocampus of mice by modulating immune responses

Ju Zeng [1,2], Bin Cheng [1,2], Yan Huang [1,2], Xiaorui Zhang [1,2], Chen Wang [1,2], Na Sun [1,2], Gang Liu [1,2], Xiaorui Cheng [1,2,*], Yongxiang Zhang [1,2,*] and Wenxia Zhou [1,2,*]

[1] Beijing Institute of Pharmacology and Toxicology, Beijing100850, China;
[2] State Key Laboratory of Toxicology and Medical Countermeasures, Beijing 100850, China;
Correspondence should be addressed to chengxr@bmi.ac.cn (X.C.); zhangyx@bmi.ac.cn (Y.Z.); zhouwx@bmi.ac.cn (W.Z.).

Temporal Order Memory Test

The exploration apparatus is an open-topped arena (50×50×50 cm) made of black plastic. Using an overhead camera and video recorder to track the mouse. The A object was square pyramid and B object was conical flask filled ink that were too heavy for the animal to displace. This task comprised two sample phases and one test phase. Before this task, each mouse was placed into apparatus for 20 min allowed to freely explore for 2 days, then returned to their home cage. In each learning phase, the mice were allowed to explore two copies of an identical object for 15 min, with a delay between the learning phase1 and 2 of 1 h. The test phase was given 6 hr after learning phase 2. During the test trial, a copy of the object A from phase 1 and a copy of object B from phase 2 were used, and the positions of the objects in the test not changed, the mice were allowed to explore for 4 min. If temporal order memory is intact, the mice will spend more time exploring the object A. The discrimination ratio was calculated as the time spent by each mouse exploring the object A divided by the total time spent exploring both objects in the test period.

The Pretreatment of LW-AFC Has No Effect on Temporal Order Memory Impairment in LPS--treated Mice

In order to know the effect of LW-AFC on cognitive change of LPS-induced mice, we chose the temporal order memory test to investigate the object discrimination ratio in LPS-treated mice. The results showed that the model group mice spent more time for exploring object B than object A (Figure S1C, D), this data suggest that the injection of LPS impaired the temporal order memory of mice. But there were no other significant differences of LW-AFC treatment.

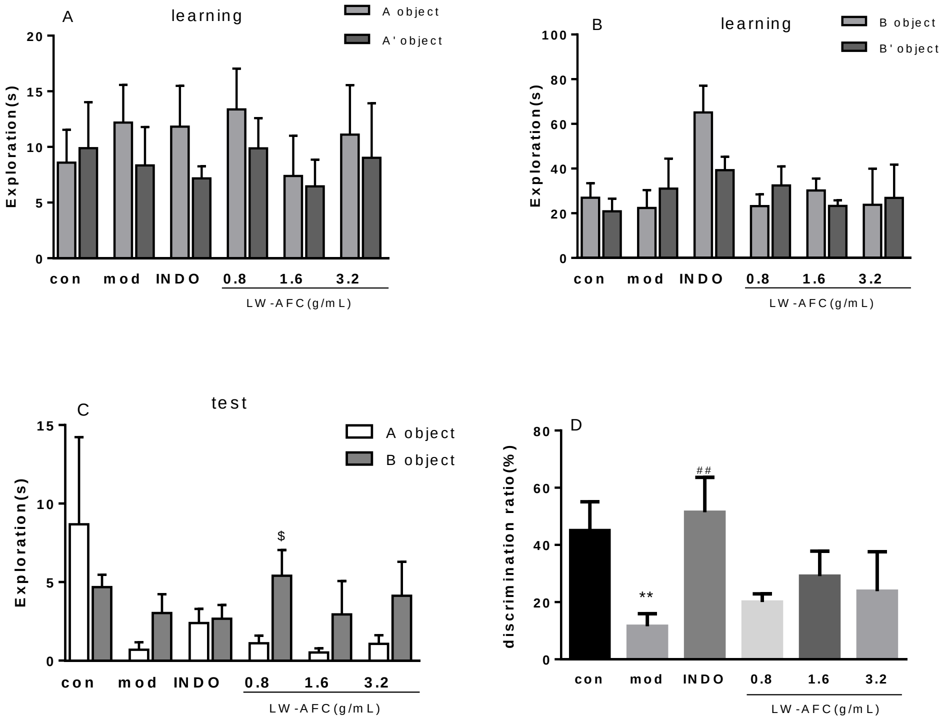

Figure S1: The effects of LW-AFC on temporal order memory test in LPS-treated mice. (A) A object exploration time of learning phase, (B) B object exploration time of learning phase, (C) the total exploration time of test, (D) the discrimination ratio of A and B object. $^{\$}p<0.05$, *vs* LW-AFC(0.8mg/kg) A object, student's *t*-test; $^{**}p<0.01$, *vs* con, Student's *t*-test; $^{\#\#}p<0.01$, *vs* mod, one-way ANOVA and Dunnett's test, mean±S.D., n=8. con, control; mod, model; INDO, indomethacin.

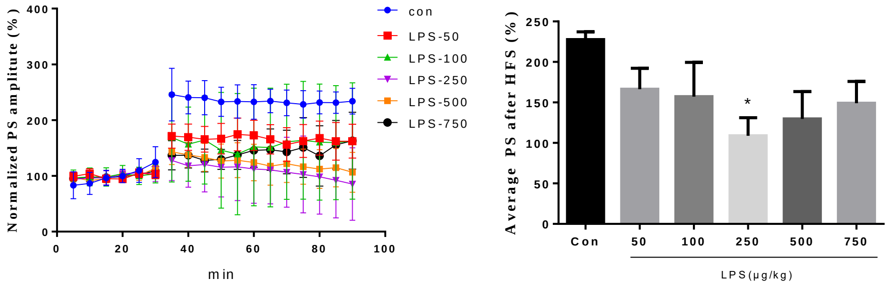

Figure S2: The effects of LPS at different doses on long-term potentiation in mice. The male Balb/c mice with the weight 19-21g were intraperitoneally injected with a single dose of lipopolysaccharide. A population spike (PS) were recorded in the hippocampal PP-DG (anterior penetrating fiber-dentate granule cell layer) position. $^{*}P<0.05$, *vs.* control, one-way ANOVA and Dunnett test. mean ± SD. n=6-7

The Extraction of LW-AFC

The herbal extracts and quality control were published on the journal of Current Alzheimer's Research [2017, 14(2): 221-238.]. The detail preparation of LW-AFC is as follows:

LW-AFC was prepared as shown in Figure 3. LW-AFC was composed of 20.3% polysaccharide fraction (LWB-B), 15.1% glycosides fraction (LWD-b) and 64.6% oligosaccharide fraction (CA-30) in the dry weight ratio. For pharmacognosy identification of LW-AFC, highperformance liquid chromatography (HPLC) method was used to analysis and control the quality of LW-AFC. For mixture of CA-30 and LWD-b, the chromatographic separation was obtained on a Diamond C18 column. The Figure 4A showed the typical HPLC fingerprint of CA-30 and LWD-b mixture. There are 17 chromatogram peaks and the No. S peak represents loganin in fingerprint of CA-30 and LWD-b mixture (Figure 4A). For LWB-B, the chromatographic separation was obtained on a Nucleosil NH2 100Å column. The Figure 4B showed the typical HPLC fingerprint of LWB-B. There are five chromatogram peaks and these five peaks represent fructose, glucose, sucrose, mannotriose and stachyose, the retention times of them were 6.260 min, 6.829 min, 8.186 min, 18.305 min and 21.506 min, respectively (Figure 4B).

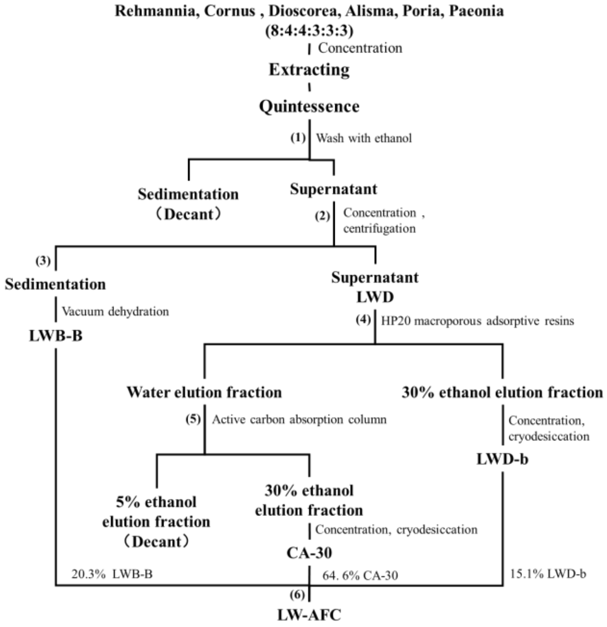

Figure S3: The schematic diagram of LW-AFC preparation.

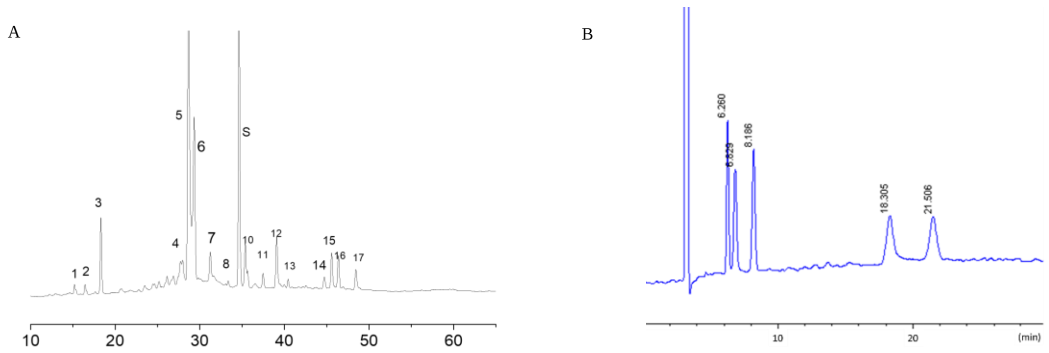

Figure S4: The HPLC fingerprint of LW-AFC. (A) The HPLC fingerprint of LWD-B and CA30 mixture. The peak S is loganin. (B) The HPLC fingerprint of LWB-B. The peak at 6.26, 6.83, 8.19, 18.30 and 21.50 min is respectively fructose, glucose, sucrose, mannotriose and stachyose.

Pretreatment of LW-AFC Modulated the Cytokines Secretion in cortex Caused by Intraperitoneal Injection of LPS

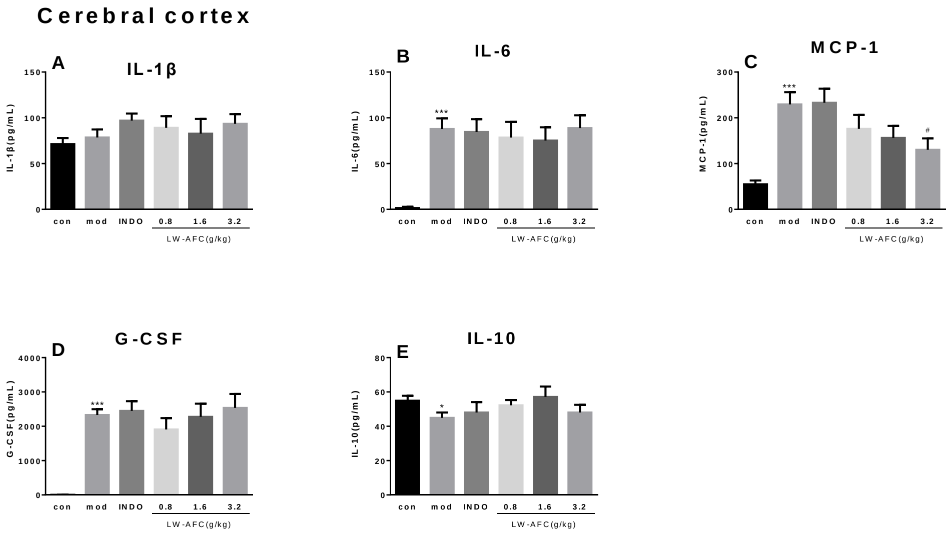

Figure S5 The effect of LW-AFC on cytokine secretion in cerebral cortex of mice with intraperitoneal injection of LPS. ****p*<0.001, **p*<0.05, *vs* con, Student's *t*-test; #*p*<0.05, vs mod, one-way ANOVA and Dunnett's test. mean±S.D., n=5-8.
